# Professional Advertisements

**Published:** 1878-09

**Authors:** 


### PROFESSIONAL ADVERTISEMENTS.

*To the Chicago Medical Journal and Examiner:*

The subject of personal advertising has recently been brought prominently to the notice of the profession. The methods, both direct and indirect, of accomplishing this are often observed, and, if carefully collated, would afford material for an interesting, possibly an instructive article. To arrange to be called out of a congregation; to drive in great haste, as if pressed with business, though perhaps sadly lacking patients; and in many other ways to convey the impression of being overwhelmed with professional work, and to appear as if possessed of a hidden reserve of knowledge and skill, have been practiced until the appliance of these devices almost takes the rank of an established art. Another method is to advertise through the columns of the secular press, or in handbills and circulars, that may be easily disseminated. In this practice a large class that the profession look upon as quacks, assume no disguise, but boldly proclaim their skill and acquirements. This method, however, is condemned by the recognized profession, and is prohibited by its code of ethics. But the ingenuity of man has always been able to devise means to evade the statutes, both of law and ethics. When the State of Maine made the selling of a glass of whisky to a thirsty sufferer a penal offense, places of business were immediately opened where a striped pig was exhibited for a consideration, and the delighted beholder went away gratuitously refreshed with an exhilarating draught. Druggists, who are conscious of the indelicacy of too directly advertising themselves, select some special article which they advertise with great diligence, the gist of the advertisement being like that of a lady's letter, in the postscript—for, at its close, the druggist's card holds a conspicuous place. There are suspicions that some members of the profession practice similar arts.

I may cite, as an example of what may justify these suspicions, the headings of certain certificates recently widely disseminated. These headings recite the names of the physicians who give the certificates, the professorships they hold in various colleges, the hospitals with which they are connected, the learned societies of which they are members, and in some cases even descend to the minutiæ of the street and number of their residence.

To these headings are attached certificates, recommending the use of certain mineral waters. This reproach rested on a number of well known physicians of the city of New York. The JOURNAL AND EXAMINER, animadverting on this occurrence at the time, said: "It is a satisfaction to us, that at no time have we had occasion to blush for any such flagrant violation of professional propriety in the West." But "let not him that girdeth on his harness boast himself as he that putteth it off." Close on the heels of this self-congratulation, the profession of Chicago has been made to feel the crimson in its cheek. An advertisement appears day after day in the most widely circulated papers of Chicago, signed by more than a dozen well-known physicians of the city, who have united in the Herculean task of informing a distressed and waiting public, of whom it may purchase a pair of spectacles. By a singular accident, most of these signers are the happy possessors of long titles; and these are carefully embodied in the advertisement. It is Professor this, or Professor that, in this or that medical college; or an attendant in some hospital. A single name comes before the public in the nakedness of a simple M. D., and with its surroundings in the advertisement, it appears like one bald head amid a group, of which all others are adorned with a profusion of hyperian locks. Few can be so blind as not to see that such advertisements are as much in the interest of the "Professors" whose names are embraced in them, as of the innocent draught of mineral water, or bit of pebble glass recommended. This is not unlike the Arab cry of the street venders of small wares. "In the name of the Prophet—figs." In the name of the "Professors"—spectacles. Such arts lack even the merit of novelty. I presume the metrorrhagic woman of the Scriptures—"which had an issue of blood twelve years, and had suffered many things of many physicians, and had spent all she had, and was nothing better, but rather grew worse," had selected in succession her many medical attendants, from having seen their advertisements in the daily prints, and been in this manner informed that each of them had the degree of A. M., M. D. and L.L. D., and was Professor in the Oriental Medical College at Jerusalem; or held a position in some hospital there established. These oriental Professors might have denied that this was an individual advertisement of their attainments, and thus a violation of the code; but they were certainly conscious that only a dull patient would fail to reason that high position is presumptive evidence of superior skill. Possibly professional advertising is now practiced as successfully as ever before; but it is sadly lacking in that display of genius which once gave it almost an air of respectability. To make this apparent we quote from some advertisements of Martin Van Butchell, a physician who practiced in London more than a century since:

Here is a recommendation of the Balm of Life, published in the *World*, Saturday, August 30, 1791.

"He that doeth well, cometh to the light."

"Old People, (efpecially Profeffors of the healing art; and fuch of their friends, as are not fond of inteftine medicines) are refpectfully informed, that MARTIN VAN BUTCHELL. (of Mount-ftreet, Grofvenor-fquare), Surgeon-Dentift, and Patentee for Spring-Bands, has invented and (himfelf) prepared, a neat, clean, pleafant-fmelling, outward SPECIFIC, for BLEEDING FUNGUS', INFLAMMATIONS, SUPPURATIONS, CARBUNCLES, ULCERATIONS, and MORTIFICATIONS!

"JOHN HUNTER, Esq. F. R. S. (of Leicefter-fquare), Surgeon-Extraordinary to the King, and Surgeon-general to his Majetfy's Forces, had a fmall bottle of it prefented to him very lately:—when he faw two Patients, and the faid SPECIFIC, moft readily applied (to their bad parts), with much fuccefs!

This—is not like any extract of Lead;—
(That palfy-producing,—noxious Metal:—)
Nor,—the King's-Evil Roufer—Mercury:—
(Dire,—predifpofing Caufe—of many Woes.—)
Nor,—Antimony;—often dangerous:—
(Nor,—the Cauftick,—Lapis Infernalis:—)
Nor,—that injurious poifon,—Arfenick:—
(Nor,—Hemlock; which, too long amus'd weak minds)
But,—ESSENCE from fine Gold;—
Sweet BALM of LIFE!
"The ROYAL COLLEGE of PHYSICIANS,
And the
Corporation of SURGEONS of London,
(Thofe proper GUARDIANS of infirm bodies,)

May fee—if they pleafe—fuch as Mr. JOHN HUNTER has with pleafure feen: on giving notice to

MARTIN VAN BUTCHELL,
"Who fends forth words of truth—and foberness."
☞ So many die—wanting the BALM of LIFE;
Aged folk—fhould ever have it by them!—
And fo ought the young:—as a PREVENTIVE,
Of what might end in—
Sad PUTRIDITY.——
Obferve,—
Strong Drinks—Make Weaknefs,—and are bad
For them that ufe
VAN BUTCHELL's Balm of Life!

The two following are designed to advertise the doctor rather than his medicines:

*BY the GRACE of GOD.*
WE—CURE—SAD—TOOTH—ACH:
In TWENTY minutes:—Without DRAWING It:
LIKEWISE, BAD RUPTURES:—VERY SPEEDILY:
With FIT Bandages:—THAT do NOT Torture.
MARTIN VAN-BUTCHELL:
SURGEON for the TEETH:—More than THIRTY Years,
"THEY—THAT—SEEK—SHALL—FIND."
*TUMORS—ON—FACES:*
Gums,—NECKS,—and ARMS of—THE faireft LADIES:

20

And Fair Gentle-Men:
STYS—upon—EYE-LIDS:
Paralytic—Strokes:—Weaknesses—of—Joints:
*BIG—VEINS—IN—MAN's—LEGS:*
FUNDAMENTAL-ills:—PROLAPSUS-ani:
*WENS and CARBUNCLES:*
Swellings—near—the—Groin:—Fistulæ-and-Piles:
CURED—without—CUTTING;
*Or SCARIFYING.*

MARTIN VAN-BUTCHELL, from his Anatomical—Chemical—Experimental—Mechanical—Medical—Philofophical—Practical—and Surgical Knowledge, is depended on, as the trueft friend to fober people, forely afflicted—with faid fad complaints.

*EMPERORS,—Princes,—DUKES, and—MARQUISES.*
The Great JOHN Hunter * Taught Me to Get first:
So we do much good:—Curing Fistulæ.
*Without Confinement, Fomentation, Rifk:*
*Injection, Poltice, Cauftic, or cutting.*
FEE is Two per Cent.—On Five Years Profit:
☞All the money down:—Before I begin.
ANANIAS, Fell!—Dead For KEEPING Back!
MARTIN VAN-BUTCHELL.

* Surgeon Extraordinary To The King:
And Surgeon General to His Forces.
THE—first MAGISTRATE
And other Sincere—Lovers of this State
Are now informed—moft refpectfully
That fome years ago, Martin Van Butchell, had an Appointment to mee
(—At Lady Hunloch's Houfe, in Stratford-Place,—)
his able teacher, John Hunter, Efq.
Who overtook him in Grofvenor-fquare, and bade him get into his Chariot:
—foon as he was feated,
*John*, faid, "What Mifchief are you about now?"
*Martin.* 'Curing the King's-Evil.'
*John.* "I can't Cure the King's-Evil."

*Martin.* 'I know you can't Cure the King's Evil:—if you could Cure the 'King's-Evil, I fhould not trouble myfelf about the King's-Evil but I want 'to do,

'What you cannot do!'

*John.* "That is right.—Do you try to get firft. (—*We know nothing, com- 'pared to what we are ignoront of*—) Make yourfelf of confequence, and then 'every body will make you of confequence; but if you don't make yourfelf 'of confequence, nobody elfe will.—I do affure you,—many are in very 'high efteem, and very full practice, that (—comparatively—) know no more 'about Healing, than *Dray-Horfes*:—they have not Powers.

"You—Try—To—Be—First!"

These advertisements are classical, and make those of more recent origin seem "stale, flat and unprofitable." The study of Van Butchell may safely be commended to all who may be tempted to seek personal fame otherwise than by doing worthy work and communicating their observations to the profession through scientific channels.

It is not pleasant to feel obliged to criticise in this manner the acts of personal and professional friends, but the interests of the profession should always stand above personal considerations, and if our code is not to be maintained, we may as well disband all our organizations, and become, at once, professional guerrillas.

E. INGALS, M. D.

---

MILWAUKEE, August 20, 1878.

*Editors Chicago Medical Journal and Examiner:*

In the issue of your journal for November of last year, you took occasion to severely censure a number of prominent Eastern physicians for displaying their names and titles in advertisements of the Hunyadi Janos mineral waters. You justly denounced it as a violation of professional propriety which representative men certainly ought to be the most scrupulous in observing.

You concluded your criticism by saying: "It is a satisfaction to us that we at no time have had occasion to blush for any such flagrant violation of professional propriety in the West."

When I read this, I experienced the pride of being a Western physician. I imagined myself and my Western brethren standing on the high pedestal of professional integrity, from which we could look down upon the Eastern crowd toiling in the dust of newspaper notoriety.

But alas! My pedestal was built of perishable stuff, and erected on quicksand! To-day it lies on the ground, a heap of dust and imagination.

Scarcely six months had passed over the country, when physicians of the Western metropolis set an example of professional impropriety far more glaring and disgraceful than the public indorsement of a mineral water by our Eastern brethren.

I allude to the advertisement of an optician, which during the past month was published on the first page of the Chicago daily and Sunday papers, and paraded the names and titles of eighteen professors in the medical colleges of Chicago as indorsers.

The Eastern physicians can say at least that they recommended a *genuine* article, which really *is* that which it claims to be.

They could form their opinion at least from experience, and an intimate knowledge of the article they recommended.

But what about the article indorsed by eighteen Chicago medical professors? Did they inquire into the previous history of this case; did they ascertain whether he was a competent, honest-dealing optician, or an impostor? It would also be pleasing to know how these eighteen professors, among whom there is not a single ophthalmic surgeon, so suddenly obtained the ability to pass an opinion on spectacles and their proper adaptation, which they generally admit they know nothing about.

To a clear minded physician whose eye can penetrate through the gauzy garment of a "professor," to the true inwardness of the knowledge and ability of a man, these indorsers appear to have made themselves ridiculous because they did not know what they were talking about, and have disgraced their names and colleges if the recommended optician should sooner or later be unmasked as a shrewd unscrupulous pretender.

I was accustomed to look to the professors as the representative men of our profession, and believed them to be the corner stones of the code of ethics. I always thought they were unusually pedantic in their professional acts lest they might soil the purity of professional dignity; and exceedingly reluctant to have their names and titles appear in daily newspapers lest somebody might suspect them of a desire to advertise themselves. I, therefore, was greatly surprised by this exhibition of bad taste, ill judgment, and imprudence on the part of our professors.

Your most sorrowful brother,

OPTIC.

